# Efficacy and mechanisms of curcumin in the treatment of osteoarthritis: A scoping review

**DOI:** 10.17305/bb.2024.11045

**Published:** 2024-12-19

**Authors:** Xiaodong Ma, Wenjian Zhao, Fan Yang, Kok-Yong Chin

**Affiliations:** 1 Department of Traditional Chinese Medicine, Universiti Tunku Abdul Rahman, Kajang, Malaysia; 2 Department of Pharmacology, Faculty of Medicine, Universiti Kebangsaan Malaysia, Cheras, Malaysia; 3 Department of Pathology, College of Basic Medicine, Xiangnan University, Chenzhou City, China; 4 School of Public Health and Laboratory Medicine, Hunan University of Medicine, Huaihua City, China; 5 Cancer Center, General Hospital of Hunan Medical University, Huaihua City, China

**Keywords:** Arthritis, curcuminoids, cartilage, chondrocytes, joint

## Abstract

Osteoarthritis (OA) is a degenerative joint disease that primarily affects the elderly worldwide. It is characterized by local inflammation, which can be targeted therapeutically using natural anti-inflammatory compounds such as curcumin. This scoping review explores the therapeutic effects and mechanisms of curcumin in OA management. A total of 50 relevant original studies published in English were selected from PubMed, Web of Science, and Scopus using specific search strings, regardless of study type. These studies demonstrated curcumin’s anti-inflammatory, protective, and anti-apoptotic effects on chondrocytes. Curcumin has been shown to stimulate chondrocyte proliferation and collagen production while inhibiting matrix metalloproteinase activity. These mechanisms contribute to curcumin’s ability to alleviate pain and improve joint function in OA patients. While the findings highlight curcumin’s potential in OA management, further research is needed to enhance its bioavailability and determine optimal formulations, dosages, and administration routes.

## Introduction

Osteoarthritis (OA) is a common and chronic degenerative joint disease characterized by progressive changes in articular cartilage degradation, subchondral bone sclerosis, osteophyte formation, angiogenesis within the subchondral bone, and joint inflammation [1]. The condition primarily impacts middle-aged and elderly individuals, with a higher prevalence in women compared to men, and its frequency increases significantly with age [2]. Approximately 303 million individuals worldwide are affected by OA, with a prevalence rate of around 37% in individuals aged 50 and above, increasing to approximately 80% in those aged 75 and older [3].

OA is associated with a high morbidity, which significantly impacts the quality of life for patients and exerts significant burdens on families and society. Common symptoms of OA include joint pain, swelling, stiffness, and limited mobility, which can progress to joint deformities and loss of function in severe cases [3, 4]. Initial symptoms of OA are typically mild but worsen gradually, impacting the ability of patients to perform daily activities and work effectively [4]. While the precise etiology of OA is not yet fully understood, it is recognized as a complex condition influenced by various factors, such as genetics, age, gender, body mass, and occupational exposures [5, 6]. Pathological processes in OA include degradation of articular cartilage, inflammation in the synovial membrane, alterations in subchondral bone structure and modifications in the joint environment [5–7]. In the pathogenesis of OA, inflammatory cytokines, including interleukin (IL)-1β, IL-6, and tumor necrosis alpha (TNF)-α, are vital in activating downstream signaling pathways, such as nuclear factor kappa B (NF-κB) and phosphatidylinositol 3-kinase (PI3K)/protein kinase B (Akt), which in turn impact chondrocyte function and viability, ultimately resulting in matrix degradation and chondrocyte apoptosis [5, 8, 9].

Therapeutic approaches for OA include pharmacotherapy, physical therapy, or surgical procedures. Pharmacological interventions typically consist of nonsteroidal anti-inflammatory drugs and chondroprotective agents to manage pain and enhance joint function [10–13]. Physical therapies, such as extracorporeal shockwave therapy and intermediate-frequency pulse electrotherapy, improve blood flow and decrease inflammation [10, 11]. In cases of severe OA, joint replacement surgery may be warranted. The use of traditional Chinese Medicine (TCM) in treating OA is on the rise. TCM treatment emphasizes restoring homeostasis through herbal remedies, acupuncture, and massage to alleviate symptoms [12, 13].

Recent advancements in the understanding of the pathological mechanisms of OA have significantly influenced the development of therapeutic strategies [14]. Research focusing on the Wnt signaling pathway, the interplay between autophagy and apoptosis and synovial inflammation has provided new opportunities for treating OA [8]. For instance, Wnt signaling activation is essential for cartilage homeostasis. However, its aberrant activation in OA leads to chondrocyte apoptosis and matrix degradation [15]. Inhibition of Wnt signaling has been shown to improve OA in clinical trials [16]. Additionally, the emergence of innovative technologies, such as gene engineering and cell-based therapies, offers potential for novel approaches to managing OA [17]. Although the etiology of OA is complex, a cure has not yet been discovered. Therefore, the current treatments primarily focus on alleviating symptoms, slowing disease progression, and improving quality of life [8, 14, 17, 18].

Curcumin, a polyphenolic compound derived from turmeric rhizomes, is well-recognized for its potent anti-inflammatory, antioxidant, and anticancer properties [19]. It inhibits the synthesis of diverse proinflammatory cytokines, neutralizes free radicals, and elicits extensive pharmacological impacts via numerous signaling cascades [20]. Additionally, curcumin exhibits neuroprotective and cardioprotective capabilities, improving cognitive functions and mitigating cardiovascular ailments [21, 22]. Research indicates that curcumin exhibits beneficial effects in the management of OA, with clinical data supporting its ability to reduce symptoms and enhance the quality of life in patients, particularly in the initial to moderate phases of the condition [19]. Considering the involvement of inflammation and antioxidants in the development of OA, curcumin may have the potential as a functional dietary component for preventing this disease [20].

This review aims to provide a comprehensive understanding of curcumin’s potential as a disease-modifying intervention for OA, potentially informing the development of novel therapeutic strategies. Since many systematic reviews and meta-analyses on the efficacy of curcumin in treating OA are available [23–27], this review will focus on the mechanisms through which curcumin may enhance joint health and mitigate the onset of OA from preclinical studies as well, potentially bridging the gap between preclinical findings and clinical applications.

## Materials and methods

This scoping review adhered to the guidelines outlined in the Preferred Reporting Items for Systematic Reviews and Meta-Analyses (PRISMA) for scoping reviews (Table S1). The scoping review methodology was specifically chosen to investigate the relationship between curcumin and OA, enabling the exploration of a diverse range of studies to comprehensively understand the current research landscape in this area.

**Table 1:** PICOS of the current review

**Item**	**Description**
Population (P)	Cellular studies Animal studies Human studies
Intervention (I)	Curcumin (oral, injection or topical application)
Comparator (C)	Placebo, vehicle, analgesics, or no treatment
Outcome (O)	Cellular studies: Chondrocyte viability and proliferation Animal studies: Cartilage or subchondral bone structure Human studies: Pain and functional scales, radiographic imaging Applicable to all studies: Expression of cartilage health, inflammatory and redox markers, molecular signaling pathway regulation
Study design (S)	All study designs

### Defining research question

The primary research question for this scoping review was: “What are the protective effects of curcumin against osteoarthritis?” The population, intervention, comparator, outcome, and study design (PICOS) criteria for studies included in this review are summarized in Table 1.

### Literature search

A systematic literature search was conducted across three databases—PubMed, Web of Science, and Scopus—up to October 2024. The search strategy utilized a combination of keywords and Boolean operators: (Osteoarthritis OR Osteoarthritides OR Osteoarthrosis OR Osteoarthroses OR “Degenerative arthritis”) AND (Curcumin OR Turmeric OR Curcuminoid).

### Literature selection

Inclusion criteria for articles were: (1) primary studies investigating the effects of curcumin on OA, and (2) studies conducted using cellular systems, animal models, or human subjects. Exclusion criteria included: (1) conference abstracts, review articles, letters to the editor, commentaries, or opinion pieces lacking original data; (2) studies that did not use pure curcumin as the treatment; and (3) publications in languages other than English.

Three authors (X.M., W.Z., and F.Y.) independently searched the databases using the specified keywords. Retrieved literature was organized using Endnote version X9 (Clarivate, Philadelphia, PA, USA). Duplicate items were identified both automatically by Endnote and manually by the researchers. The authors screened the titles and abstracts of each article, retrieving full texts for further evaluation only when deemed relevant. Decisions regarding inclusion were made through mutual agreement among the three authors. In cases of disagreement, input from K.Y.C. was sought.

### Data extraction and synthesis

Relevant data were systematically extracted by the authors (X.M., W.Z., and F.Y.) using structured data extraction sheets. Extracted information included authorship, publication year, study methodology, dosage, treatment duration, results, and limitations. The data were synthesized qualitatively and categorized into thematic areas for analysis.

## Results

### Selection of literature

**Figure 1.**
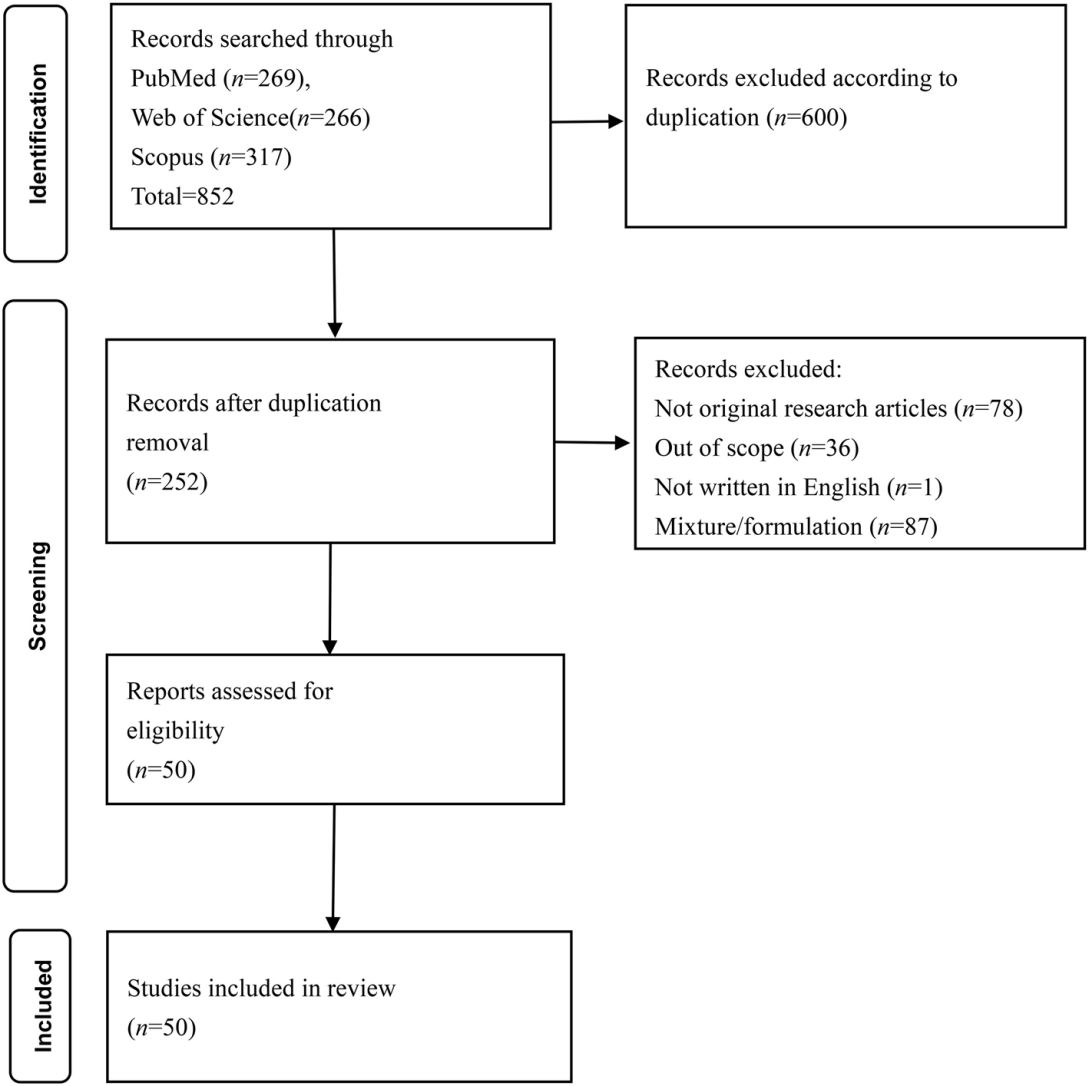
**PRISMA flowchart on the article selection process.** PRISMA: Preferred Reporting Items for Systematic Reviews and Meta-Analyses.

The initial literature search yielded 852 articles from the PubMed (*n* ═ 269), Web of Science (*n* ═ 266), and Scopus (*n* ═ 317) databases. After eliminating duplicated records (*n* ═ 600), 252 articles were included for further screening. Subsequently, 202 articles were excluded from the study because they did not meet the inclusion criteria (not original research article ═ 78; not within the scope ═ 36; not written in English ═ 1, not using pure curcumin ═ 87). Ultimately, 50 articles that met all the inclusion criteria were included in the analysis and summarized in Tables 2–4. Figure 1 displays the flowchart illustrating the selection process.

**Table 2:** Cellular studies regarding the effects of curcumin on joint health

**No.**	**Authors (years)**	**Cell culture studies**	**Major findings (Changes vs. negative control)**			**Conclusion**
		**Study design**	**Indices decreased**	**Indices increased**	**Indices unchanged**	
1	Schulze-Tanzil et al. (2004) [71]	Cell: Primary human chondrocytes Induction: Induced by IL-1β (10 ng/mL for 0, 4, 8, 12 or 24 h) Treatment: Curcumin (50 µM) Control: Negative: No treatment Positive: No	MMP-3 Nuclear translocation of NF-κB	Collagen type-II	NA	Curcumin protects chondrocytes from the catabolic effects of IL-1 β, such as MMP-3 upregulation, and relieves cytokine-induced suppression of matrix protein synthesis.
2	Shakibaei et al. (2005) [72]	Cell: Primary human chondrocytes Induction: 10 ng/mL IL-1β or TNF-α alone for 24h Treatment: Curcumin (50 µM) Control: Negative: No treatment Positive: No	COX-2 and MMP-9 Phosphorylation levels of NF-κB p65 Nuclear translocation of activated phospho-p65 IκBα phosphorylation, ubiquitination and degradation p-Akt	Collagen type II β1-integrin (CD29)	NA	Curcumin suppresses NF-κB mediated IL-1β/TNF-α catabolic signaling pathways in chondrocytes.
3	Shakibaei et al. (2007) [32]	Cell: Primary human chondrocytes Induction: Induced by 10 ng/mL IL-1β for 0, 5, 15, 30 min Treatment: Curcumin (50 µM) Control: Negative: No treatment Positive: No	Morphological degenerative features Activated caspase-3	Collagen type-II Microvilli-like cytoplasmic processes β1-integrin synthesis	NA	Curcumin exerts anti-apoptotic and anti-catabolic effects on IL-1β-stimulated articular chondrocytes.
4	Jackson et al. (2006) [73]	Cell: Primary bovine chondrocyte Induction: IL-1(20 ng/mL) Treatment: Curcumin (0.1, 1, 10 µM) Control: Negative: No treatment Positive: No	MMP-1, MMP-3	Proteoglycan	NA	Curcumin suppresses MMP expression and increased proteoglycan expression.
5	Lev-Ari et al. (2006) [74]	Cell: Human OA synovial adherent cells Induction: No Treatment: Curcumin (20 µM) Control: Negative: No treatment Positive: Celecoxib	Growth of OA synovial adherent cells PGE2 synthesis	Apoptosis	COX-1 COX-2	Curcumin inhibits the growth of OA synovial adherent cells and enhances the induction of apoptosis by celecoxib.
6	Toegel et al. (2008) [75]	Cell: Immortalized C-28/I2 chondrocytes Induction: IL-1β (10 ng/mL) Treatment: Curcumin (5 µM, 50 µM) Control: Negative: No treatment Positive: No	NA	Area of dead cells (50 µM) COL2/COL1 ratios (50 µM) Expression of MMP3, ADAMTS4 (50 µM)	Total cell count and proliferative activity (5 µM) AGC or COL2 (5 µM)	Curcumin is not effective at lower concentrations. It damaged the cells at higher concentrations.
7	Clutterbuck et al. (2009) [76]	Model: Cartilage explant model from equine joints Induction: 10 ng/mL or 25 ng/mL IL-1β Treatment: 100 µmol/L curcumin Control: Negative: No treatment Positive: NA	GAG release induced by IL-1β	NA	NA	Curcumin at 100 µmol/L significantly reduces IL-1β-stimulated GAG release in cartilage explants, suggesting a potential anti-inflammatory effect in an *in vitro* model of cartilage inflammation.
8	Mathy-Hartert et al. (2009) [77]	Cell: Human chondrocytes Induction: Human recombinant IL-1β (10^-11^M) Treatment: Curcumin (5, 10, 15 and 20 µM) Control: Negative: No treatment Positive: No	NO, PGE2, IL-6, and IL-8 (15 and 20 µM) MMP-3, the molar ratio of MMP-3/TIMP-1	NA	IL-6 and IL-8 (5 and 10 µM) TIMP-1 Aggrecan	Curcumin inhibits the production of inflammatory and catabolic mediators by chondrocytes.
9	Buhrmann et al. (2010) [78]	Cell: Canine MSCs and primary chondrocytes Induction: IL-1β (10 ng/mL) Treatment: 5 µM curcumin for 4h Control: Negative: No treatment Positive: NA	Activation of caspase-3, COX-2, NF-κB, Iκ-Bα phosphorylation	Collagen type II, cartilage-specific proteoglycans, β1-integrin, ERK1/2, Sox-9, MAPKinase signaling	NA	Curcumin may help establish a microenvironment which antagonizes proinflammatory cytokines, thus facilitating chondrogenesis of MSC-like progenitor cells.
10	Clutterbuck et al. (2013) [34]	Cell: Equine articular chondrocytes Induction: IL-1β (10 ng/mL) Treatment: Curcumin (3 µM–100 µM) Control: Negative: No treatment Positive: Carprofen (100 µg/mL)	PGE2, MMP-3, proteoglycan release	Cell viability (≤ 25 µM curcumin)	NA	Curcumin’s mechanism of action may involve inhibiting NF-κB, which leads to reduced production of inflammatory mediators and catabolic enzymes.
11	Yang et al. (2013) [79]	Cell: Rabbit articular chondrocytes Induction: Advanced glycation end products (100 µg/mL) Treatment: Curcumin (10–50 µM) Control: Negative: No treatment Positive: NA	TNF-α mRNA and protein expression, MMP-13 mRNA expression ROS production, MDA level IκBα phosphorylation and degradation, NF-κB activation (p65 nuclear translocation)	CAT and SOD activity, expression of TNF-α and MMP-13	NA	Curcumin inhibits the inflammatory activity against AGE-induced activation of rabbit chondrocytes by suppressing ROS production and NF-κB activation.
12	Wang et al. (2017) [80]	Cell: Rat articular chondrocytes Induction: IL-1β (10 ng/mL) Treatment: Curcumin (50 µM) Control: Negative: No treatment Positive: NA	Expression of MMP-13 Inhibition of NF-κB activation (IκBα phosphorylation and p65/RelA nuclear translocation)	Expression of type II collagen, chondrocyte proliferation	NA	Curcumin protects articular chondrocytes via inhibition of NF-κB signaling.
13	Li et al. (2017) [66]	Cell: Primary rat chondrocytes Induction: 10 ng/mL IL-1β Treatment: Curcumin (10 µM) Control: NA Negative: 3-Methyladenine (10 nM) Positive: Rapamycin (10 µM)	Number of apoptosis chondrocytes Active caspase 3 Autophagosomes	Bcl-2 autophagic vacuoles, LC3-II and beclin-1 p-ERK1/2	NA	Curcumin suppressed apoptosis and inflammatory signaling through its actions on the ERK1/2-induced autophagy in chondrocytes.
14	Cao et al. (2017) [81]	Cell: C3H10T1/2 mesenchymal stem cells Induction of chondrogenesis: Chondrogenic differentiation medium containing TGF-β3 Treatment: 1 µM curcumin for 14, 21, 28 days Control: Negative: No treatment Positive: NA	Hypertrophic markers (Runx2, Col10α1, Mmp13), Gli2, NICD, Hey1	NA	Chondrogenic markers (Sox9, Col2a1)	Curcumin inhibits chondrocyte hypertrophy in MSCs without affecting chondrogenic differentiation. This is due to the downregulation of the IHH and Notch signaling pathways, which are crucial for chondrocyte hypertrophic differentiation.
15	Zhao et al. (2018) [82]	Cell: Rabbit articular chondrocytes Induction: 1 mM SNP Treatment: Curcumin (5-20 µM) Control: Unstimulated controls Negative: SNP	Apoptosis, the loss/disruption of Δ Ψm, NO production, Bax expression, cleaved caspase-3 expression, MMP-13 expression	Cell viability, SOD activity, GSH-Px activity, Expression of type II collagen and Bcl-2	NA	Curcumin suppresses SNP-insulted chondrocyte apoptosis via the mitochondrial-dependent pathway.
16	Feng et al. (2019)* [36]	Cell: Human OA synovial cells Induction: Tert-Butyl hydroperoxide (20 µM) Treatment: Curcumin (20 µM) Control: NA Negative: No treatment Positive: No	Chondrocyte apoptosis Proapoptotic protein levels (cleaved caspase3 and cleaved PARP) CHOP, GRP78 and ATF4 p-PERK, p-eIF2α	Viability of chondrocytes Expression of COLII and Bcl-2 SIRT1	NA	Curcumin inhibits ER stress and the related PERK-eIF2α-ATF4-CHOP signaling pathway.
17	Zeng et al. (2019) [33]	Cell: Human OA synovial cells Induction: No Treatment: Curcumin (0, 5, 10, 20, 40 µmol/L) Control: NA Negative: No treatment Positive: No	Cell viability MMP3 expression	Fibronectin 1 Collagen III	NA	Curcumin downregulated the expression of MMP3, promoted apoptosis and inhibited proliferation of synovial cells, and alleviated OA inflammatory response.
18	Jiang et al. (2020)* [63]	Cell: Human TMJ chondrocytes Induction: IL-1β (10 ng/mL) Treatment: Curcumin (20 and 40 µM) Control: NA Negative: No treatment Positive: No	iNOS, COX-2 ROS levels MMP-1, MMP-3, MMP-9 and MMP-13	COL2α1 and ACAN expression Nrf2 and p-Nrf2 levels mRNA levels of HO-1 SOD2, NQO-1, GCLC NQO-1 and HO-1 protein expression	NA	Curcumin activates the ROS/Nrf2/HO-1-SOD2-NQO-1-GCLC signaling axis to reduce inflammation and oxidative stress in TMJ chondrocytes.
19	Qiu et al. (2020) [83]	Cell: Primary chondrocytes (species not declared) Induction: IL-1β treatment Treatment: Exosomes derived from MSCs treated with or without curcumin Control: Negative: No treatment Positive: NA	Apoptosis DNA methylation of miR-143 and miR-124 promoters NF-κB, ROCK1, TLR9 expression	Cell viability Expression of exosome-specific proteins (CD9, CD63, CD81) Expression of miR-143 and miR-124	NA	Curcumin protects primary chondrocytes by increasing the expression of miR-143 and miR-124, which target key proteins in OA pathogenesis. This effect was mediated through decreased DNA methylation of the miRNA promoters, leading to increased miRNA expression and subsequent downregulation of NF-κB and ROCK1.
20	Zhang et al. (2021) [68]	Cell: Primary rat chondrocytes Induction: No Treatment: Curcumin (8 µM) Control: Negative: No treatment Positive: No	NA	Proliferation and migration of articular chondrocytes Gene coding for COL2α1, aggrecan, and Sox9 expression	NA	Curcumin enhances the BMSCs function for the proliferation and migration of articular chondrocytes, and anabolic gene expression of extracellular matrix in articular chondrocytes.
21	Buhrmann et al. (2021) [65]	Cell: Primary human chondrocytes Induction: 3-dimensional (3D) osteoarthritic environment (OA-EN) model (consisting of fibroblasts, T-lymphocytes, and 3D-alginate with chondrocytes to better mimic the heterogeneous pro-inflammatory OA-EN *in vivo*) Treatment: Curcumin (1, 2, 5, 10 µM) Control: Negative: No treatment Positive: No	Chondrocyte number, viability Cox-2, MMP-9, cleaved-caspase-3 DNA binding of p65-NF-κB Inflammation and chondrocyte apoptosis	p65-NF-κB Sox9, Sox9-p65-NF-κB complex Type II collagen and cartilage-specific proteoglycans (CSPG)	NA	Curcumin suppresses inflammation in OA-EN via modulating NF-κB-Sox9 coupling and maintains homeostasis in OA by balancing chondrocyte survival and inflammatory responses.
22	Chen et al. (2021) [64]	Cell: Primary rat chondrocytes Induction: IL-1β (10 ng/mL) Treatment: Curcumin (1.25, 2.5, 5, 10, 20 µM) Control: Negative: No treatment Positive: No	LDH release in chondrocytes Collapse of the Δ ψm Chondrocyte apoptosis Cleaved-PARP, cleaved-caspase-3, and cleaved-caspase-9 Phosphorylation levels of NF-κB p65, GSK-3β, and β-catenin NF-κB p65 activity	Proportion of autolysosomes Levels of LC3-II and Beclin-1	NA	Curcumin suppresses apoptosis of IL-1β-induced primary rat articular chondrocytes. The NF-κB pathway plays a key role in the protective effects of curcumin against chondrocyte apoptosis induced by IL-1β.
23	Wang et al. (2021)* [41]	Cell: Murine knee chondrocytes Induction: IL-1β (10 ng/mL) Treatment: Curcumin (10, 20 and 50 µM) Control: Negative: No treatment Positive: NA	Expression of catabolic genes (MMP9, ADAMTS5) Protein expression of iNOS and COX2, Activation of NF-κB/HIF-2α pathway (the ratios of pNF-κB/NF-κB, pIκB-α/IκB-α, and HIF-2α/GAPDH, nuclear translocation of NF-κB and HIF-2α)	Cell viability and proliferation Expression of anabolic genes (SOX9, aggrecan, Col2α)	NA	Curcumin protects murine knee chondrocytes treated with IL-1β by blocking the NF-κB/HIF-2α signaling pathway.
24	Jin et al. (2022)* [30]	Cell: Primary rat chondrocytes Induction: IL-1β (10 ng/mL) Treatment: Curcumin (10 µM) Control: Negative: No treatment Positive: No	ROS and Ca^2+^ levels MMP13 and IL-1β	Mitochondria Δ ψm and intercellular ATP levels Markers of mitophagy (PINK1, Parkin, LC3B, P62, and Beclin1) and collagen II p-AMPK Co-localization of mitophagy markers (LC3B and COXIV)	NA	Curcumin exerts chondroprotective effects against OA by activating mitophagy via the AMPK/PINK1/Parkin pathway.
25	Yuan et al. (2022) [31]	Cell: Chondrocytes HC-a and C28/I2 cells Induction: No Treatment: Curcumin (0.1, 1, 10 µM) Control: Negative: No treatment Positive: No	IL-6, IL-1β, and IL-8 Chondrocyte apoptosis	Protein levels of Wnt3a, Wnt5a, and β-catenin	NA	Curcumin activates the Wnt/β-catenin signaling pathway to inhibit chondrocytes’ inflammatory reaction and apoptosis.
26	Zhou et al. (2023) [84]	Cell: Mouse primary chondrocytes Induction: 3 µM erastin for 24 h Treatment: Curcumin (5 µM) Control: Negative: No treatment Positive: NA	LDH, MDA, ROS, Fe2^+^ contents Ferroptosis-related proteins (ACSL4, TFR1), erastin-induced chondrocyte ferroptosis	Chondrocyte viability, SOD, GPx Expression of ferroptosis-related proteins (SLC7A11, GPx4, FTH1), Nrf2 expression	N/A	Curcumin enhances the ferroptosis resistance of chondrocytes by activating Nrf2.
27	Deng et al. (2024) [85]	Cell: Human articular chondrocytes Induction: 10 ng/mL IL-1β for 24 h Treatment: Curcumin (10 µM) Control: Negative: No treatment Positive: Meloxicam (10 µM)	IL-1β, IL-6 and TNF-α levels D-glucuronic acid, D-xylitol, imidazole-4-acetaldehyde levels Pyruvic acid, l-methionine, pyruvate, l-alanine, glutaminylserine, 1-isothiocyanato-6-(methylsulfinyl)hexane, D-xylose, urocanic acid, imidazole-4-acetaldehyde, methylimidazole acetaldehyde and L-methionine levels mRNA expression levels of DCXR, DHDH, GPAT3, PLPP4, AOC2, CTH, GALNT3, CDO1, POC1B-GALNT4, PIP4K2C, INPP5J, ITPKA, ITPKB and ISYNA1	2-phospho-D-glyceric acid, 2-phospho-d-glyceric acid, D-2,3-dihydroxypropanoic acid levels mRNA expression levels of cystathionine-β-synthase (CBS), PSAT1, MAOA, DGAT2, PLPP2, ALDH3B1, GALNT5, GALNT15, PLCH2	NA	Curcumin have therapeutic potential in the management of OA by targeting CBS, PSAT1, MAOA and other crucial proteins.
28	Wang et al. (2024) [86]	Cell: Chondrocytes Induction: 2 mM SNP stimulation for 24 h Treatment: Curcumin (5 µM) Control: Negative: No treatment Positive: NA	Pro-inflammatory cytokines (IL-6, COX-2, MMP-9, MMP-1) Apoptosis markers (caspase-3) Oxidative stress markers (ROS) p38/MAPK signaling pathway (ASK1, MEKK3, p-p38, p-MAPK, p-pkcδ)	Chondrocyte viability, mitochondrial membrane potential, Bcl-2	NA	Curcumin inhibits SNP-induced chondrocyte apoptosis by modulating the p38/MAPK signaling pathway, upregulating Bcl-2 expression and downregulating caspase-3 expression.

**Table 3:** Animal studies regarding the effects of curcumin supplementation on joint health

**No.**	**Authors (years)**	**Cell culture studies**	**Major findings (Changes vs. negative control)**			**Conclusion**
		**Study design**	**Indices decreased**	**Indices increased**	**Indices unchanged**	
1	Nonose et al. (2014) [47]	Animal: Male Sprague-Dawley rats (8 weeks old) Induction: 0.05 mL zymosan (1 mg/50 µL) in both knee joints Treatment: Gastric gavage with curcumin every 6 h for 48 h at a dose of 100 mg/kg Control: Negative: Intra-articular infiltration with saline in both knees and gastric gavage with corn oil every 6 h for 48 h Positive: Gastric gavage with corn oil every 6 h for 48 h	Numbers of neutrophils (6 h after the induction)	NA	Numbers of neutrophils (12, 24 and 48 h after the induction)	Curcumin reduces inflammation in the first 6 h after experimentally zymosan-induced arthritis.
2	Zhang et al. (2016) [44]	Animal: Male C57BL/6 mice (5-6 months old) Induction: Surgically transecting the medial meniscal-tibial ligament (MMTL) in the right hind limb, leading to destabilisation of the medial meniscus (DMM) Treatment: Oral administration of 50 mg/kg curcumin dissolved in corn oil Control: Negative: Oral gavage of corn oil Positive: NA	Cartilage fibrillation OARSI score Synovitis and subchondral plate thickness	NA	OA-related pain (von Frey testing, distance travelled and hindlimb rearing)	Curcumin significantly reduces OA disease progression but did not significantly affect OA pain relief.
3	Sun et al. (2017) [38]	Animal: Male C57BL/6 mice (age not mentioned) Induction: MMTL transection to generate DMM Treatment: Daily intraperitoneally injected curcumin at 50 mM after surgery Control: Negative: Daily intraperitoneally injected DMSO after surgery Positive: NA	OA scores IL-1β, IFN-γ, IL-17A, IL-18, TNF-α and VCAM1 in cartilage tissue samples	NA	NA	Curcumin significantly reduces OA progression in the DMM model. Curcumin suppresses mRNA expression of proinflammatory mediators in the arthrodial cartilage of mice with OA.
4	Yan et al. (2019) [39]	Animal: Male Sprague-Dawley rats (age not mentioned) Induction: Transection of the anterior cruciate ligaments (ACL) in the right knees Treatment: 50 µL of curcumin at the concentration of 1 mg/mL by intra-articular injection once a week for 8 weeks Control: Negative: Injection of 50 µL saline into the knee joints Positive: NA	Percentage of TUNEL-positive apoptotic chondrocytes Inflammatory cell infiltration and synovial lining cell layer IL-1β and TNF-α contents TLR4 expression NF-κB expression	Chondrocytes	NA	Curcumin exerts anti-inflammatory effect on OA, at least in part, by suppressing the TLR4 pathway.
5	Feng et al. (2019)* [36]	Animal: Male Sprague-Dawley rats (8-week-old) Induction: Transection of the right ACL Treatment: Curcumin once daily for 8 weeks by intraperitoneal injection (50 mg/kg or 150 mg/kg) Control: Negative: 0.9% Normal saline via intravenous injection Positive: NA	Sclerosis of the cartilage surface, narrowing of the space of the knee joint, destruction, erosion and lesions of articular cartilage OARSI score Apoptosis of chondrocytes Cleaved caspase3 and CHOP levels	Chondrocytes in tissue sections Proteoglycan levels	NA	Curcumin attenuates knee joint degradation and decreased chondrocyte apoptosis in rats with OA. The protection is through activation of SIRT1 and inhibition of ER stress.
6	Zhang et al. (2019) [37]	Animal: Female Sprague–Dawley rats (age not mentioned) Induction: Intra-articular injection of MIA (1 mg/50 µL saline) at the right knee joint Treatment: Intraperitoneal injection of 200 mg/kg curcumin for 14 consecutive days Control: Negative: Intraperitoneal injection of normal saline Positive: NA	Diameter of joint Infiltration of inflammatory cells Mankin’s scores Expression of IL-6, IL-1β and TNF-α Expression of MyD88, p-IκBα, NF-κB	The degree of smoothness of the joint surface and cell arrangement Chondrocytes in tissue sections Paw withdrawal threshold	NA	Curcumin blocks TLR4/NF-κB signal pathway and reduces inflammation levels to prevent knee wound in OA rats.
7	Nicoliche et al. (2020) [46]	Animal: Male Wistar rats (12 weeks of age) Induction: Single intra-articular injection of zymosan (1 mg/50 µL saline) Treatment: Oral administration of 50 mg/kg/day curcumin for 60 days Control: Negative: Oral administration of corn oil Positive: NA	Indian hedgehog (IHH) expression in the middle layer Articular cartilage thickness of the middle and deep layers	Col2 expression in all articular layers SOX-5 expression in the surface and deep cartilage layers Chondrocyte number in the surface and middle layers	IHH expression in the surface and deep cartilage layers Chondrocyte number in the deep layer	Curcumin protects cartilage by increasing IHH, Col2 and SOX-5 expression and the number of chondrocytes.
8	Jiang et al. (2020)* [63]	Animal: Male Sprague-Dawley rats (8 weeks old) Induction: Injection of complete Freund’s adjuvant (CFA) into the TMJ cavity Treatment: Weekly injections of 40 µM curcumin in NS for 4 weeks Control: Negative: Weekly injections of normal saline containing 0.1% DMSO for 4 weeks Positive: NA	Cartilage erosion, proteoglycan loss Levels of iNOS, COX-2, IL-1β, MMP-9 and MMP-13	Nuclear Nrf2 expression	NA	Curcumin suppresses inflammation at TMJ via iNOS and Nrf2 pathway.
9	Park et al. (2020) [45]	Animal: Male Wistar rats Induction: MIA injection Treatment: Theracurmin at 500, 1300, or 2600 mg/kg for 5 weeks Control: Negative: Saline Positive: 100 mg/kg/day Celebrex	Weight-bearing imbalance, Mankin scores, cartilage damage, chondrocyte number, Expression of nitrotyrosine, TNF-α, phosphorylated NF-κB, cleaved caspase-3	NA	NA	Theracurmin protects MIA-induced OA by suppressing inflammation signaling in rats.
10	Nakahata et al. (2021) [40]	Animal: Male Wistar rats Induction: DMM surgery Treatment: 30 mg/mL curcumin monoglucuronide (tBP1901) intra-articular injections for 10 weeks Control: Negative: Saline Positive: N/A	Synovial inflammation (at weeks 1 and 2), TNF-α expression in articular cartilage (at week 6), Articular cartilage damage SB plate thickening and plate perforation, osteophyte formation (at week 10)	Type II collagen expression in articular cartilage (at week 10)	NA	TBP1901 intra-articular injections suppressed synovial inflammation in the acute phase of OA in DMM rats. In the chronic phase, TBP1901 suppresses articular cartilage damage and regulates SB plate changes.
11	Wang et al. (2021)* [41]	Animal: C57BL/6 male mice Induction: DMM Treatment: Curcumin 50 mg/kg/day curcumin in corn oil for 6 weeks Control: Negative: Corn oil only Positive: NA	NF-κB positive cells and HIF-2α positive cells	Cartilage matrix staining and the rough articular surface, ICRS II score PCNA immuno-reactive cells in the joint region	NA	Curcumin improves the integrity of the articular cartilage partially by suppressing the NF-κB/HIF-2α signaling pathway.
12	Jin et al. (2022) [30]	Animal: Male Sprague-Dawley rats (7 weeks old) Induction: 0.2 mg/50 µL MIA-intra-articular injection Treatment: 0.5% curcumin via intravenous injection for 4 weeks Control: Negative: 0.9% Normal saline via intravenous injection Positive: NA	Cartilage degeneration Mankin and OARSI scores MMP13 expression	Chondrocyte proliferation Levels of collagen II, PINK1, P62, Beclin-1, and LC3B	NA	Curcumin enhanced collagen anabolism and reduced inflammatory catabolism in articular cartilage.
13	Saber et al. (2023) [43]	Animal: Male Wistar rats (10-12 weeks old) Induction: 100 µL MIA-intra-articular injection at the right knee Treatment: Oral administration of curcumin at 200 mg/kg for 35 days Control: Negative: Oral administration of normal saline Positive: NA	Serum CRP and knee COMP levels HDAC3 in the knee joint Levels of MMP1 and MMP13 NF-κB, IL-1β, and TNF-α	Rotarod time miR-130a in the knee joint PPAR-γ in the knee joint	Paw withdrawal threshold	Curcumin exhibits the potential to mitigate joint swelling and pain, while also demonstrating an ability to modulate inflammation.
14	Hua Ye et al. (2024) [42]	Animal: Male Sprague Dawley rats (age?) Induction: Hulth surgery Treatment: 150 mg/kg/day curcumin for 35 days Control: Negative: 0.5% sodium carboxymethyl cellulose solution (2 mL/day) Positive: NA	Severity of cartilage damage Expression of atrogin-1 and MuRF-1 in the quadriceps femoris muscle Autophagy (decreased LC3II/LC3I ratio and increased p62 expression) ROS levels, SOD2 acetylation	The gait parameters (maximum contact area, mean intensity and stand) SIRT3 expression, SOD2 activity Quadriceps femoris muscle cross-sectional area	NA	Curcumin promotes quadriceps femoris muscle regeneration in rats with knee OA. It may play a protective role *in vivo* by clearing ROS through SIRT3-SOD2 pathway to inhibit autophagy.
15	Ding et al. (2024) [87]	Animal: Male C57BL/6 mice (10-week-old) Induction: DMM Treatment: 25 mg/kg/day curcumin for 8 weeks Control: Negative: 0.5% sodium carboxymethyl cellulose solution (2 mL/day) Positive: NA	Mankin score of cartilage SB TRAP-positive cell number NF-κB, p-JNK, RANKL-positive cell number	Tibial SB bone volume, trabecular thickness, trabecular number, connectivity density	NA	Curcumin reduced SB sclerosis by suppressing osteoclast formation via NF-κB/JNK signaling.
16	Wang et al. (2024) [86]	Cell: Chondrocytes Induction: 2 mM SNP stimulation for 24h Treatment: Curcumin(5µM) Control: Negative: No treatment Positive: NA	Inflammatory markers (IL-6, COX-2, MMP-9, MMP-1) Apoptosis markers (caspase-3) Oxidative stress markers (ROS) p38/MAPK signaling pathway (ASK1,MEKK3,p-p38,p-MAPK, p-pkcδ)	Chondrocyte viability, Mitochondrial membrane potential Bcl-2	N/A	Curcumin inhibits SNP-induced chondrocyte apoptosis by modulating the p38/MAPK signaling pathway, upregulating Bcl-2 expression and downregulating caspase-3 expression.

**Table 4:** Clinical studies regarding the effects of curcumin on joint health

**No.**	**Authors (years)**	**Study design**	**Study Design**		**Findings**		**Conclusion**
			**Subjects’ characteristics**	**Treatment**	**Major outcomes**	**Adverse events**	
1	Panahi et al. (2014) [48]	Double-blinded RCT	Degenerative primary knee OA with mild-to-moderate severity. Bilateral OA Age: < 80 years	Curcumin (1500 mg/day in 3 divided doses) for 6 weeks	WOMAC: Improved global score, pain score, and physical function score, no improvement in stiffness score. Pain: Decreased LPFI and VAS scores	Not mentioned	Curcumin reduces pain and improves joint function in patients with OA.
2	Nakagawa et al. (2014) [49]	Double-blinded RCT	Diagnosed with grade II or III OA based on Kellgren-Lawrence criteria Age: 40 and 80 years	Theracurmin (6 capsule/day × 30 mg of curcumin) for 8 weeks	VAS scores: Reduced compared to the placebo group, especially patients with an initial score <0.15. JKOM and JOA scores: No significant differences compared to the placebo. Analgesic use: The number of patients requiring NSAIDs was reduced	Significant adverse events were not observed. A limited number of patients exhibited minor biochemical alterations	Theracumin reduces pain and the demand for NSAIDs in OA patients.
3	Rahimnia et al. (2015) [54]	Double-blinded RCT	Mild to moderate degenerative and bilateral primary knee OA Not indicated for joint replacement surgery Not using corticosteroids ≥ 10 mg/day or any IA injection for the last 3 months	Curcuminoids (1500 mg/day divided into 3 doses) for 6 weeks	WOMAC, VAS & LPFI: Significantly improved over placebo control	The incidence of adverse events did not differ between the treatment and the placebo groups	Curcuminoids are safe and effective in improving OA symptoms in patients.
4	Panahi et al. (2016) [53]	RCT	Mild to moderate primary knee OA Age: <80 years	Curcumin (1500 mg/day divided into 3 doses) for 6 weeks Piperine (16 mg/day) co-administered to improve bioavailability	Serum SOD activity: Increased in the treatment group but decreased in the control group. Serum GSH level: Increased in the treatment group, but not different compared to control. Serum MDA level: Reduced in both groups but was more pronounced in the treatment group	Not mentioned	Curcumin improves redox status in patients with OA effectively.
5	Shep et al. (2019) [50]	Open-labelled RCT	Diagnosed with knee OA for three months Did not require anti-inflammatory agents Age: 38–65 years Initial VAS > 4	Curcumin (1500 mg/day divided into 3 doses) for 28 days Control group: Diclofenac (50 mg diclofenac × 2/day)	VAS and KOOS: Reduced in both groups without significant intergroup differences. No patients who required H2-receptor antagonists	The incidence of adverse events was significantly lower in the curcumin group compared to the diclofenac group. Curcumin treatment was noted to reduce episodes of abdominal distension and weight loss	Curcumin is as effective as diclofenac in OA management and is a safer option.
6	Henrotin et al. (2019) [55]	Double-blinded RCT (multicentre, three arms)	Diagnosed with symptomatic knee OA for ≥ 3 months Confirmed radiographically (grade II or III OA based on Kellgren-Lawrence criteria) VAS > 4 Age: 45-80 years	Low dose: Biologically optimized turmeric extract (BCL) (2 capsules) + 1 placebo capsule, twice daily for 3 months. High dose: Biologically optimized turmeric extract (BCL) (3 capsules) Each capsule contained 46.67 mg of turmeric rhizome extract	Pain relief & PGADA enhancement: Improved with time, but no significant difference among different treatment modalities. Paracetamol use: Reduced in the high-dose group. NSAIDS use: Reduced in the low-dose group. Serum Coll2-1: Significant reduction at months 1 and 3 with treatment	The incidence of adverse events was significantly higher in the high-dose group compared to both the low-dose group and the placebo group. Abdominal discomfort and diarrhoea were identified as the most frequently reported adverse events	BCL improves functionality and relieves pain in patients.
7	Calderón-Pérez et al. (2020) [52]	RCT	Mild to moderate knee OA WOMAC score: 6-10 Age: 18–65 years	B-Turmactive group: 500 mg of TEs and 19.5 mg of curcumin complex for 1 week BY group: 200 mg of inactivated yeast and 164 mg of excipients	Pain: The B-Turmactive group demonstrated a more pronounced decrease in pain associated with walking up and down stairs. The overall pain assessment indicated that both treatments significantly alleviated discomfort at 3 days and 1 week, with the B-Turmactive group experiencing less pain after one week. hs-CRP level: Significantly reduced in the B-Turmactive group	No adverse effect	B-Turmactive exhibits a pronounced analgesic effect on knee OA and is concomitant with a reduction in inflammation.
8	Hashemzadet al. (2020) [51]	RCT	Diagnosed with primary knee OA Kellgren-Lawrence grade II and III Age: 45 and 65 years, Disease duration: 12 to 48 months	Nanocurcumin 40 mg every 12 h daily for 6 weeks	The curcumin group exhibited a significantly greater mean change in Pain index, physical function index and WOMAC total score: The Curcumin group showed significant improvement, even after subgroup analysis	Not mentioned	Nanocurcumin improves various domains of WOMAC.
9	Atabaki et al. (2020) [88]	Double-blinded RCT	Women Aged 40-55 years Diagnosed with knee OA Kellgren Lawrence grade II or III are VAS ≥ 5	Sinacurcumin® 80 mg/day for 3 months All patients were prescribed diclofenac sodium 50 mg/day	CD19+CD267+ B cells: Significant reduction with curcumin Treg cells: Significant increase Geometric mean fluorescence intensity of FOXP3: Increased with curcumin	No complications or adverse events were noted	Sinacurcumin® regulates the functionality of T cells and B cells.
10	Jamali et al. (2020) [89]	Double-blinded RCT	Older adults within the community Age: ≥ 60 years	Curcumin ointment (1.5 mL) was applied to the knee areas 3 times daily (morning, evening and before bedtime) for 6 weeks	Knee pain intensity: Reduced with curcumin ointment. By the end of week 6, the curcumin group had significantly lower pain intensity than the placebo group	Serious adverse events were not observed. Some patients reported mild cutaneous irritation reactions, including erythema, oedema, pruritus, etc.	Curcumin ointment demonstrates a significant analgesic effect in individuals diagnosed with knee OA.
11	Nakagawet al. (2020) [90]	Open-labelled prospective study	Primary medial or lateral type knee OA Age: ≥ 40 years Kellgren-Lawrence grade II, III, or IV	Theracurmin 6 capsules/day, total 180 mg curcumin, for 6 months	VAS, JKOM, JOA scores: Significant improvement in all patients compared to their pre-treatment values	Five patients withdrew from the study due to minor adverse effects. No significant adverse effects of Theracurmin treatment were observed	Theracurmin demonstrates favourable effectiveness and safety across diverse forms of knee OA.

### Study characteristics

This scoping review includes 50 studies published between 2004 and 2024, which investigate the effectiveness and underlying mechanisms of curcumin in treating OA. The review encompasses a range of *in vitro* and *in vivo* studies, utilizing diverse cell types and animal models to evaluate the potential therapeutic impact of curcumin on OA. Of note, five preclinical studies adopted both *in vitro* and *in vivo* approaches. Human trials investigating the clinical efficacy of curcumin in patients with OA are also included.

*In vitro* studies used primary chondrocytes from humans, rats, and cows, human osteoarthritic synoviocytes, chondrocyte cell lines HC-a and C28/I2, and mesenchymal stem cells for differentiation experiment. These cells were exposed to proinflammatory cytokines (IL-1β), oxidative stressors (tert-butyl hydroperoxide), and TNF-α to mimic OA-related inflammation and oxidative stress. Curcumin was given in concentrations of 1.25–50 µM to evaluate its protective effects on chondrocyte function, with treatment durations tailored to each study’s goals, typically lasting from 24 h to several weeks. These studies assessed curcumin’s impact on chondrocyte activities, showing it inhibited apoptosis, enhanced autophagy, reduced inflammatory cytokines, and modulated metalloproteinase (MMP) expression. Curcumin also activated key cellular pathways for chondroprotection, including AMPK/PINK1/Parkin for mitophagy, NF-κB signaling, and Wnt/β-catenin.

Fifteen *in vivo* studies were performed using male Wistar or Sprague–Dawley rats (10–12 weeks old) and C57BL/6 mice (5–6 months old). OA was induced through methods like intra-articular mono-iodoacetate (MIA) injections (50–100 µL), anterior cruciate ligament (ACL) transection, and medial meniscus destabilization. Subjects received curcumin doses of 50–200 mg/kg via oral gavage, intraperitoneal, or intra-articular injection for 35–60 days. Curcumin’s efficacy was evaluated through serum levels of C-reactive protein and cartilage oligomeric matrix protein, levels of histone deacetylase 3, MMP1, and MMP13, and inflammatory markers NF-κB, IL-1β, and TNF-α. Motor function and cartilage protection were evaluated through knee joint rotation time, miR-130a, and PPAR-γ. Curcumin’s impact on cartilage integrity, inflammation, and pain behaviors was measured using histological, biochemical, and molecular assessments.

Eleven human clinical studies have assessed the efficacy and safety of curcumin for knee OA, employing various designs like randomized controlled trials and open-label studies. Participants were mainly adults with mild to moderate knee OA, with some studies targeting specific age groups or genders, and different inclusion criteria regarding age, disease severity, and exclusion of other inflammatory joint conditions were applied. Curcumin was given in capsules, ointments, and alongside other therapies, with daily doses between 500 and 1500 mg for six weeks to six months. Outcomes measured included pain intensity via the visual analog scale (VAS) and functional capacity assessed by the Western Ontario and McMaster Universities Osteoarthritis Index (WOMAC) and Japanese Knee Osteoarthritis Measure (JKOM). Some studies also evaluated systemic oxidative stress markers and the need for extra pain medications.

### Results from *in vitro* studies

Articular chondrocytes represent the principal cellular constituents of articular cartilage tissue, with their core function being the synthesis and maintenance of the extracellular matrix (ECM), which is predominantly composed of collagen type II alpha 1 chain (Col2α1) and aggrecan (ACAN). These molecules provide essential mechanical support and elasticity to the cartilage, constituting critical determinants of joint health. *In vitro*, articular chondrocytes are commonly utilized to emulate the articular cartilage milieu, thereby facilitating the study of disease progression and evaluation of potential therapeutic interventions [28]. Furthermore, human articular chondrocytes maintain the cartilage’s structural integrity and functional capacity. They play a pivotal role in the pathogenesis of OA, wherein chondrocyte apoptosis contributes significantly to cartilage degeneration and impairment of joint function [29].

Curcumin has been demonstrated to modulate various signaling pathways involved in the survival and functionality of chondrocytes. Specifically, curcumin activates the AMPK/PINK1/Parkin pathway, facilitating mitophagy and consequently mitigating mitochondrial damage and oxidative stress in chondrocytes [30]. Furthermore, curcumin’s activation of the Wnt/β-catenin signaling pathway is associated with the inhibition of chondrocyte apoptosis and the enhancement of anabolic processes, including the synthesis of collagen type II and ACAN [31].

Additionally, it is noteworthy that curcumin has been identified as an inhibitor of the NF-κB signaling pathway, which is frequently hyperactivated in OA and plays a significant role in promoting inflammation and chondrocyte catabolic activities [32]. Through the suppression of NF-κB, curcumin diminishes the expression of inflammatory cytokines and matrix-degrading enzymes, including MMP-3 and MMP-9, which are recognized for their roles in ECM degradation [33]. Furthermore, the anti-inflammatory properties of curcumin are substantiated by its capacity to inhibit the production of prostaglandin E2 (PGE2), a critical mediator of inflammation, and to enhance the expression of anti-inflammatory molecules [34]. These combined effects on inflammatory pathways indicate that curcumin may possess therapeutic potential in maintaining chondrocyte function and mitigating the progression of OA.

**Figure 2.**
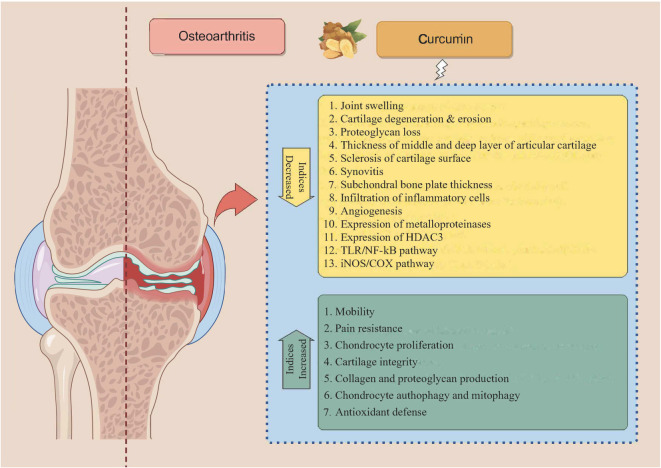
**The mechanisms of curcumin in protecting joint health.** Curcumin activates AMP-activated protein kinase, mitophagy, NF-kB, Wnt and Sirtuin 1 signaling pathways in chondrocytes, thereby upregulating the anabolic processes and downregulating the catabolic processes.

The mechanisms of curcumin in protecting joint health are summarized in Figure 2.

### Results from *in vivo* studies

OA induction models can be broadly classified into surgical and non-surgical categories. Surgical models replicate OA-like conditions by manipulating joint physiology through surgical procedures. Traditional methods involve resecting ACL, posterior cruciate ligaments, medial collateral ligaments, and medial menisci in animals. However, modified approaches often simplify these processes. For example, in canine early OA models, transection of the ACL alone is sufficient to create an early-stage OA model [35].

Curcumin has shown therapeutic potential in various surgically induced OA models. Feng et al. [36] demonstrated that post-surgical curcumin administration in rats mitigated cartilage surface hardening and joint space narrowing. It also reduced Osteoarthritis Research Society International (OARSI) scores, alleviated chondrocyte and proteoglycan loss, and inhibited OA progression in a dose-dependent manner. Similarly, Zhang and Zeng [37] found that curcumin treatment lowered OARSI scores in rat models, reduced Safranin O staining loss, cartilage fibrosis, and synovitis, and improved subchondral bone plate thickness.

Sun et al. [38] reported that curcumin significantly reduced histopathological OA scores in murine models, using a maximum scoring method to assess severity. Yan et al. [39] further observed that intra-articular curcumin preserved the cartilage matrix, maintained the structural integrity of cartilage tissue, and improved cartilage lesions caused by ACL transection (ACLT) or ACLT combined with lipopolysaccharide administration. In a study by Nakahata et al. [40], intra-articular administration of 30 mg/mL curcumin monoglucuronide (tBP1901) in rats with destabilized medial meniscus (DMM)-induced OA reduced acute-phase synovial inflammation and cartilage damage while regulating subchondral bone changes in chronic post-traumatic OA. Wang et al. [41] discovered that a six-week oral curcumin treatment enhanced articular cartilage integrity in DMM-induced mice by inhibiting the NF-κB/HIF-2α pathway. Ye et al. [42] used Hulth surgery to establish knee OA models and found that a 35-day curcumin feeding regimen supported quadriceps muscle regeneration by reducing reactive oxygen species (ROS) via the sirtuin 3 and superoxide dismutase (SOD) 2 pathway.

Non-surgical models induce OA through joint immobilization, forced motion, or chemical agents. Chemical induction methods, such as intra-articular injections of papain, hyaluronidase, collagenase, or iodoacetic acid (MIA), are widely used. Studies by Saber et al. [43] and Zhang et al. [43, 44] reported that curcumin reduced Mankin scores, knee swelling, and improved pain thresholds and joint mobility in MIA-induced OA models. Jin et al. [30] further showed that curcumin significantly reduced Mankin and OARSI scores, attenuated cartilage degeneration, and promoted chondrocyte proliferation. Park et al. [45] treated MIA-induced OA in male Wistar rats with Theracurmin® for five weeks, resulting in improved weight-bearing balance, lower Mankin scores, reduced cartilage damage, and suppression of nitrotyrosine, TNF-α, phosphorylated NF-κB, and cleaved caspase-3 expression.

Other studies using yeast polysaccharides as an OA-inducing agent highlighted curcumin’s effects on cartilage. Nicoliche et al. [46] and Nonose et al. [47] found that curcumin reduced cartilage superficial and middle-layer thickness but increased chondrocyte density in these layers. The number of deep-layer chondrocytes remained unaffected. Notably, curcumin alleviated joint inflammation within 6 h of administration but showed no significant anti-inflammatory effects after 12–24 h, potentially due to its low oral bioavailability and rapid systemic metabolism.

**Figure 3.**
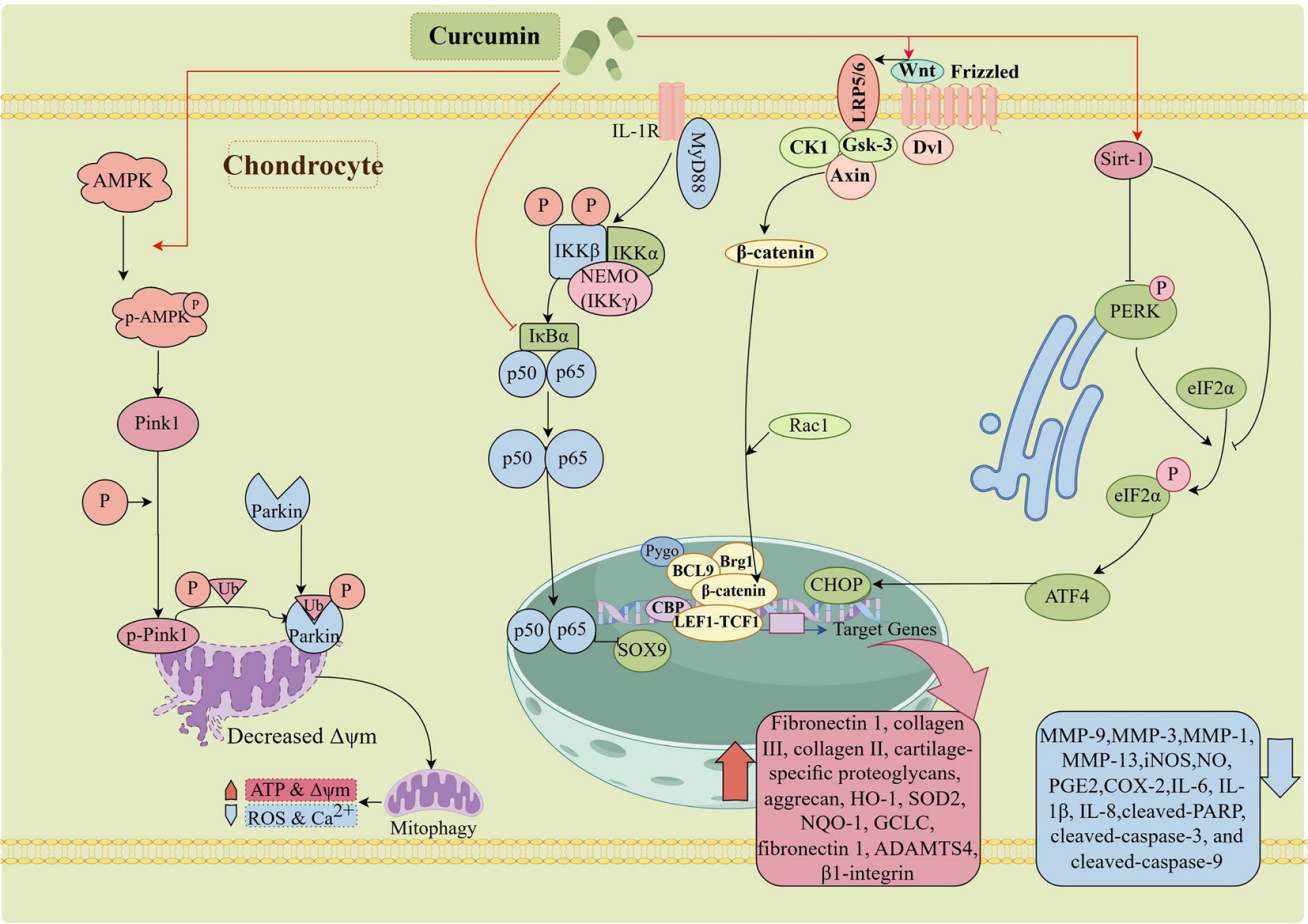
**The joint protecting effects of curcumin as demonstrated in animal models**. Curcumin protects against osteoarthritis by reducing inflammatory signaling, improving antioxidant defence and chondrocyte autophagy and mitophagy. Thus, joint structural integrity is preserved in the supplemented animals.

The joint-protective effects of curcumin across various animal OA models are summarized in Figure 3.

### Results from clinical studies

The selected studies were randomized controlled trials (RCTs) with sample sizes ranging from 40 to 85 participants, primarily employing double-blinded designs to enhance reliability. Curcumin dosages varied from 500 mg to 1500 mg and were administered via oral capsules, ointments, or bio-optimized turmeric extracts over treatment periods spanning three days to six months.

Most studies demonstrated that curcumin significantly reduced pain and improved function in OA patients compared to placebo, as evidenced by Visual Analog Scale (VAS) and Western Ontario and McMaster Universities Osteoarthritis Index (WOMAC) scores. For example, Panahi et al. [48] found that curcumin supplementation improved WOMAC global, pain, and physical function scores, though stiffness scores remained unchanged. Similarly, Nakagawa et al. [49] reported that patients receiving Theracurmin® experienced notably lower VAS scores, indicating reduced pain. Shep et al. [50] observed that curcumin was as effective as diclofenac for pain relief, with additional benefits of reducing bloating and aiding weight loss. Hashemzadeh et al. [51] reported that nanocurcumin significantly improved WOMAC scores, particularly in pain and physical function domains, compared to placebo.

Regarding its mechanism of action, curcumin’s antioxidant effects were highlighted by Panahi et al. [48], which showed increased serum SOD activity and reduced malondialdehyde (MDA) levels, reflecting improved redox status. Calderón-Pérez et al. [52] demonstrated curcumin’s anti-inflammatory effects, evidenced by a significant decrease in high-sensitivity C-reactive protein (hsCRP) levels in patients treated with B-Turmactive, a curcumin-based product.

From a safety perspective, most studies reported that adverse events in the curcumin groups were similar to or fewer than those in the placebo groups [48, 53, 54]. The standard oral dosage of 500–1500 mg daily was generally well tolerated, with long-term studies (over six months) showing sustained effectiveness and few serious adverse events. However, Henrotin et al. [55] noted a higher incidence of abdominal discomfort and diarrhea among high-dose curcumin users, suggesting that elevated doses should be approached with caution.

## Discussion

OA is a dynamic disease process characterized by pathophysiological changes involving all components of the joint tissue microenvironment, resulting from complex pathological reactions within the osteoarticular milieu [3, 4]. During the early stages of OA, the primary pathological alterations consist of cartilage injury, microcracks, and abnormal bone remodeling [56]. Healthy articular cartilage is encapsulated by an ECM composed of water, type II collagen, and glycoproteins, which collectively maintain its structure and function [57]. However, as age advances or under mechanical stress, chondrocytes may undergo inflammatory responses and apoptosis, leading to degradation of the cartilaginous matrix [2]. As OA progresses further into its intermediate and late stages, the pathology primarily manifests as subchondral bone neovascularisation, neural invasion into the cartilage, sclerosis of the subchondral bone, and osteophyte formation [2, 4, 5]. Angiogenesis and nerve growth often coexist, with new blood vessel formation, altering the hypoxic environment within chondrocytes, disrupting the balance between synthesis and degradation, and accelerating cartilage damage [5].

Chondrocytes play multifaceted roles in the pathogenesis of OA [58]. Firstly, they maintain the structural integrity and functionality of articular cartilage by synthesizing and maintaining the ECM [59]. When injured or subjected to inflammatory stimuli, chondrocytes release inflammatory mediators and matrix-degrading enzymes, such as MMPs and A disintegrin and metalloproteinase with thrombospondin, contributing to further matrix degradation and subsequent cartilage degeneration [60]. Moreover, chondrocytes undergoing oxidative stress may undergo apoptosis via pathways like PI3K/Akt, exacerbating cartilage damage [61]. Thus, chondrocytes are central players in OA development, with their injury and dysfunction contributing to matrix degradation and joint inflammation and pain, underscoring the importance of chondrocyte protection and repair strategies as potential key treatments for OA [60, 62].

Curcumin, a natural compound extracted from turmeric rhizomes, has attracted significant attention in the fields of OA prevention and therapy due to its unique pharmacological activities and broad biological effects [20]. In OA treatment, curcumin exerts various actions, including alleviation of pain, antioxidants, anti-inflammatory and anti-apoptotic, regulation of critical signaling pathways, and promotion of cartilage tissue repair [19, 20].

In terms of its antioxidant properties, curcumin exhibits outstanding performance in the OA environment by inhibiting the Nrf2/ARE signaling pathway, thereby enhancing the activity of endogenous antioxidant enzymes like SOD, effectively reducing ROS production and suppressing oxidative stress-induced damage to chondrocytes [63]. Furthermore, it activates the AMPK/PINK1/Parkin-mediated mitochondrial autophagy pathway, facilitating the clearance of damaged mitochondria and preserving normal chondrocyte function, thereby slowing down OA progression [30].

Regarding its anti-inflammatory role, curcumin potently suppresses multiple proinflammatory cytokines, such as TNF-α, IL-1β, and IL-6 [64]. It blocks the Toll-like receptor 4 (TLR4)/MyD88/NF-κB signaling cascade, decreasing the expression levels of inflammatory cytokines, reducing the infiltration of inflammatory cells into the joint and the release of inflammatory mediators, thus alleviating joint inflammation [64, 65]. Additionally, curcumin synergizes with nonsteroidal anti-inflammatory drugs like celecoxib to further inhibit the activation of NF-κB and subsequent generation of proinflammatory mediators and MMPs, preventing further destruction of the cartilage matrix [65].

Concerning the regulation of chondrocyte apoptosis, curcumin upregulates SIRT1 expression, inhibits protein kinase RNA-like ER kinase (PERK)- eukaryotic translation initiation factor 2 alpha (eIF2α)-C/EBP homologous protein (CHOP) pathway activation and triggers autophagy through ERK1/2 signaling, thereby effectively inhibiting the programmed cell death process in chondrocytes [66]. Moreover, curcumin also protects chondrocytes against apoptosis triggered by various stress factors by inhibiting pathways, such as JAK2/STAT3 and p38MAPK [30].

Regarding cartilage repair, curcumin increases the expression of protective molecules specific to cartilage, such as type II collagen and β1 integrin, while concurrently inhibiting the production of matrix-degrading enzymes like MMPs, thereby contributing to the maintenance of the integrity of the cartilage matrix and promoting the regeneration and repair of articular cartilage [67, 68]. Coupled with appropriate physical therapies, such as swimming exercises, curcumin application can further improve the inflammatory state of the synovial membrane, reduce joint swelling and pain, and facilitate the recovery of joint function in OA patients [69].

Pain is the main symptom of OA, severely affecting daily life, mobility, and emotional well-being, and can lead to economic strain and poor treatment adherence. Effective pain management is essential to enhance patients’ quality of life. Curcumin has been found effective in alleviating pain in knee OA patients, as evidenced by multiple clinical studies [48–51].

### Perspectives

Curcumin’s multifaceted mechanisms of action position it as a promising agent for both the prevention and treatment of OA, demonstrating significant potential and practical efficacy. However, several critical issues remain to be addressed to harness its therapeutic benefits fully.

Primarily, the low bioavailability of curcumin significantly constrains its clinical utility. Future research endeavors should prioritize the development of innovative preparation and delivery systems designed to enhance curcumin’s bioavailability. This can be achieved through the application of advanced technologies, such as nanotechnology, liposomal encapsulation, and novel drug carriers, thereby optimizing its absorption and utilization within the body [70].

Secondly, optimizing the dosing regimen and delivery mechanism is essential. Currently, the optimal dosage and administration schedule for curcumin in OA treatment are not well established. There is a pressing need for large-scale clinical trials to determine the most efficacious and safe dosing parameters, which will facilitate the development of more precise treatment protocols, improving patient outcomes and adherence. Currently, a daily oral dose of 1500 mg of curcumin is reported to be efficacious against OA and is relatively safe.

Thirdly, while the short-term benefits of curcumin in OA patients have been documented, there needs to be more data regarding its long-term efficacy and safety profile. Future investigations must include long-term clinical trials to comprehensively assess curcumin’s sustained effects and potential adverse reactions across diverse patient populations, ensuring its clinical applicability is both effective and safe.

Fourthly, the synergistic potential of curcumin, when combined with other therapeutic modalities, such as TCM and physical therapy, warrants exploration. Future studies should investigate the integrated use of curcumin alongside alternative treatments, evaluating their collective impact on OA management, thereby offering patients a broader spectrum of treatment options.

Moreover, although current research has elucidated some of curcumin’s mechanisms of action—namely, its anti-inflammatory and antioxidant properties and regulatory effects on apoptosis and signaling pathways—these mechanisms require further scrutiny. Future studies should concentrate on elucidating the precise cellular and molecular mechanisms underlying curcumin’s therapeutic effects, particularly focusing on its influence on chondrocyte proliferation, inflammatory cytokine levels, collagen synthesis, and matrix metalloproteinase activities. Of note, molecular and structural changes in OA differ at the early stage and the advanced stage, and between weight-bearing and non-weight-bearing joints. The mechanism of action of curcumin at different stages of OA and different types of joints should be investigated. This deeper understanding will guide clinical application and maximize curcumin’s therapeutic potential.

Lastly, the differential response to curcumin among various patient demographics, including age, gender, and genetic predispositions, necessitates investigation. Future research should scrutinize the efficacy and safety of curcumin across diverse populations, evaluating its application potential in distinct patient subgroups to enable personalized treatment strategies.

Through the diligent pursuit of these research avenues, the full therapeutic potential of curcumin in treating OA can be unlocked, paving the way for safer and more efficacious treatment alternatives for patients.

### Limitations

This review identifies several constraints. Firstly, the diversity in research methodologies, doses, and modes of curcumin administration among the incorporated studies hampers the direct comparisons of findings. Furthermore, the exclusion of non-English literature and studies not employing pure curcumin may have resulted in the exclusion of pertinent data. Grey literature was not collected in this review, thus subjecting the content to publication bias. Subsequent investigations should fill these knowledge gaps to offer more comprehensive and widely applicable findings regarding curcumin’s therapeutic capabilities in the management of OA.

## Conclusion

Curcumin, through its diverse mechanisms, including anti-inflammatory, antioxidant, apoptosis regulatory, anti-angiogenesis, and immune-modulatory pathways, holds substantial promise as a therapeutic agent for OA. Future studies should focus on optimizing curcumin’s administration strategies, assessing its long-term efficacy, and exploring its combination with other treatment modalities, with the goal of providing OA patients with safer and more effective therapeutic options.

## Supplemental data

**Table S1:** Preferred reporting items for Systematic reviews and Meta-Analyses extension for Scoping Reviews (PRISMA-ScR) Checklist

**Section**	**Item**	**PRISMA-ScR checklist item**	**Reported on page #**
Title			
Title	1	Identify the report as a scoping review.	1
Abstract			
Structured summary	2	Provide a structured summary that includes (as applicable): background, objectives, eligibility criteria, sources of evidence, charting methods, results, and conclusions that relate to the review questions and objectives.	2
Introduction			
Rationale	3	Describe the rationale for the review in the context of what is already known. Explain why the review questions/objectives lend themselves to a scoping review approach.	1-3
Objectives	4	Provide an explicit statement of the questions and objectives being addressed with reference to their key elements (e.g., population or participants, concepts, and context) or other relevant key elements used to conceptualize the review questions and/or objectives.	5
Methods			
Protocol and registration	5	Indicate whether a review protocol exists; state if and where it can be accessed (e.g., a web address); and if available, provide registration information, including the registration number.	NA
Eligibility criteria	6	Specify characteristics of the sources of evidence used as eligibility criteria (e.g., years considered, language, and publication status), and provide a rationale.	6
Information sources*	7	Describe all information sources in the search (e.g., databases with dates of coverage and contact with authors to identify additional sources), as well as the date the most recent search was executed.	5
Search	8	Present the full electronic search strategy for at least 1 database, including any limits used, such that it could be repeated.	5
Selection of sources of evidence†	9	State the process for selecting sources of evidence (i.e., screening and eligibility) included in the scoping review.	6
Data charting process‡	10	Describe the methods of charting data from the included sources of evidence (e.g., calibrated forms or forms that have been tested by the team before their use, and whether data charting was done independently or in duplicate) and any processes for obtaining and confirming data from investigators.	6
Data items	11	List and define all variables for which data were sought and any assumptions and simplifications made.	6
Critical appraisal of individual sources of evidence§	12	If done, provide a rationale for conducting a critical appraisal of included sources of evidence; describe the methods used and how this information was used in any data synthesis (if appropriate).	NA
Synthesis of results	13	Describe the methods of handling and summarising the data that were charted.	6
Results			
Selection of sources of evidence	14	Give numbers of sources of evidence screened, assessed for eligibility, and included in the review, with reasons for exclusions at each stage, ideally using a flow diagram.	6-7
Characteristics of sources of evidence	15	For each source of evidence, present characteristics for which data were charted and provide the citations.	7-8
Critical appraisal within sources of evidence	16	If done, present data on critical appraisal of included sources of evidence (see item 12).	NA
Results of individual sources of evidence	17	For each included source of evidence, present the relevant data that were charted that relate to the review questions and objectives.	8-13
Synthesis of results	18	Summarize and/or present the charting results as they relate to the review questions and objectives.	Tables 2-4
Discussion			
Summary of evidence	19	Summarize the main results (including an overview of concepts, themes, and types of evidence available), link to the review questions and objectives, and consider the relevance to key groups.	13-16
Limitations	20	Discuss the limitations of the scoping review process.	18
Conclusions	21	Provide a general interpretation of the results with respect to the review questions and objectives, as well as potential implications and/or next steps.	18
Funding			
Funding	22	Describe sources of funding for the included sources of evidence, as well as sources of funding for the scoping review. Describe the role of the funders of the scoping review.	1

This is a standard PRISMA-ScR checklist available for download from https://www.prisma-statement.org/scoping. It is attached here to ensure the current review conforms to the PRISMA-ScR guidelines. JBI: Joanna Briggs Institute; PRISMA-ScR: Preferred Reporting Items for Systematic reviews and Meta-Analyses extension for Scoping Reviews. * Where *sources of evidence* (see second footnote) are compiled from, such as bibliographic databases, social media platforms, and Web sites. † A more inclusive/heterogeneous term used to account for the different types of evidence or data sources (e.g., quantitative and/or qualitative research, expert opinion, and policy documents) that may be eligible in a scoping review as opposed to only studies. This is not to be confused with *information sources* (see first footnote). ‡ The frameworks by Arksey and O’Malley (6) and Levac and colleagues (7) and the JBI guidance (4, 5) refer to the process of data extraction in a scoping review as data charting. § The process of systematically examining research evidence to assess its validity, results, and relevance before using it to inform a decision. This term is used for items 12 and 19 instead of “risk of bias” (which is more applicable to systematic reviews of interventions) to include and acknowledge the various sources of evidence that may be used in a scoping review (e.g., quantitative and/or qualitative research, expert opinion, and policy document). *From:* Tricco AC, Lillie E, Zarin W, O’Brien KK, Colquhoun H, Levac D, et al. PRISMA Extension for Scoping Reviews (PRISMAScR): Checklist and Explanation. Ann Intern Med. 2018;169:467–473. doi: 10.7326/M18-0850.
